# Muscle quality during cancer treatment and associations with health-related quality of life

**DOI:** 10.1007/s11136-026-04175-2

**Published:** 2026-02-12

**Authors:** Jacob L. Rodrigues, Nathaniel S. O’Connell, Arnethea L. Sutton, Kathryn J. Ruddy, Kathryn E. Weaver, Glenn J. Lesser, Bonnie Ky, Ralph B. D’Agostino, W. Gregory Hundley, Moriah P. Bellissimo

**Affiliations:** 1 Department of Internal Medicine, Division of Cardiology, Pauley Heart Center, Virginia Commonwealth University School of Medicine, Richmond, VA USA; 2 Department of Biostatistics and Data Science, Wake Forest University School of Medicine, Winston-Salem, NC USA; 3 Department of Kinesiology and Health Sciences, Virginia Commonwealth University, Richmond, VA USA; 4 Division of Medical Oncology, Mayo Clinic, Rochester, MN USA; 5 Department of Social Sciences and Health Policy, Wake Forest University School of Medicine, Winston-Salem, NC USA; 6 Department of Internal Medicine, Section on Hematology and Oncology, Wake Forest University School of Medicine, Winston-Salem, NC USA; 7 Department of Medicine, Perelman School of Medicine at the University of Pennsylvania, Philadelphia, PA USA

**Keywords:** Breast cancer, Skeletal muscle, Intermuscular fat, Fatigue

## Abstract

**Purpose:**

Breast cancer (BC) survivors report declines in health-related quality of life (HRQoL), including worsening physical function and fatigue levels. It is uncertain if these changes are associated with muscle quality. This study estimated changes in muscle quality and HRQoL in BC participants over 24 months of treatment and tested associations with muscle quality and HRQoL.

**Methods:**

Women (n = 149, 50.3 ± 10.7 years) diagnosed with stage I-III BC (PREVENT-WF-98213) reported HRQoL via Patient-Reported Outcomes Measurement Information System (PROMIS) surveys at baseline prior to cancer treatment and at 6-months and 24-months follow up from treatment initiation. Paraspinal intermuscular fat (IMF) and skeletal muscle (SM) were determined by magnetic resonance imaging, and the IMF:SM ratio was calculated to estimate muscle quality. Analyses included linear mixed-effects models adjusting for study group (placebo/statin), age, race, and body mass index.

**Results:**

HRQoL declined from baseline to 6-months but returned to baseline levels by 24-months. Muscle quality worsened from baseline through 24-months manifested by an increase in IMF. Individuals with better muscle quality at baseline reported declines in HRQoL from baseline to 6-months (mean difference [MD] = − 0.12 ± 0.03, *p* < 0.001), whereas participants with poor baseline muscle quality did not (MD = − 0.06 ± 0.003, *p* = 0.14). An increase in IMF:SM over 24-months trended towards worse physical function (− 8.76 ± 5.79, *p* = 0.132).

**Conclusion:**

Muscle quality worsened over 24-months and trended toward worse physical function at 24-months follow-up. From initial declines during treatment, participants reported recovered HRQoL by 24-months. Further work in a larger cohort is needed to confirm associations with muscle quality and physical function.

**Supplementary Information:**

The online version contains supplementary material available at 10.1007/s11136-026-04175-2.

## Introduction

Over the last three decades, breast cancer (BC) mortality has declined by 43%. Now, about 80% of BC survivors live at least 10 years after diagnosis and become long-term survivors [1]. However, there are rising concerns about cancer therapy-associated late adverse health effects, including reduced health-related quality of life (HRQoL). Cancer survivors report declines in HRQoL domains from the point of diagnosis, through treatment, and post-treatment [2–6]. Importantly, poor HRQoL may increase BC survivors' mortality risk [7]. The physical symptoms related to cancer and associated treatments, including limited physical function and increased fatigue, are some of the most recognized and studied components of HRQoL [8]. Due to the growing population of BC survivors, it is a high public health priority to identify factors that may influence HRQoL to provide targets for interventions that attenuate the negative effects of cancer and cancer therapy.

Muscle quality may be one factor underlying declines in HRQoL. Poor muscle quality is defined as a high amount of fat within and around a muscle group (i.e., intermuscular fat, IMF) relative to the amount of muscle. Poor muscle quality is associated with increased inflammation, metabolic dysregulation, and may impact the health and function of skeletal muscle by impairing oxygen uptake and mitochondrial oxidative capacity [9–13]. Increased IMF is a predictor for survival and risk for cardiovascular disease among cancer survivors [14–16]. Yet, longitudinal changes in muscle quality within a population of cancer survivors have not been described, and the role of muscle quality on HRQoL in patients receiving cancer treatment is unknown. Understanding these associations can inform efforts to prevent declines or improve long-term recovery of HRQoL in cancer survivors.

The aim of this study was to report changes in muscle quality and HRQoL over 24 months of cancer treatment and determine if declines in muscle quality were associated with worse HRQoL. We hypothesized that poor muscle quality would be associated with lower physical function and greater fatigue.

## Methods

**Fig. 1:**
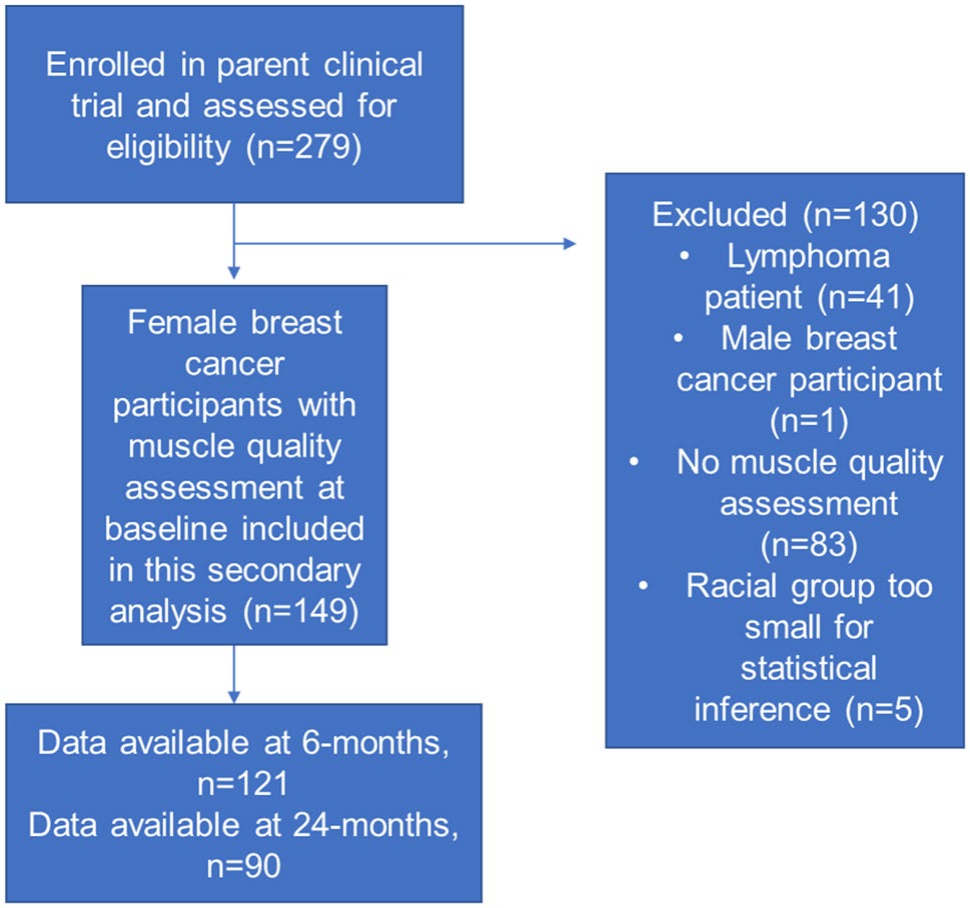
Flow chart of included study participants. There were 74 participants with muscle quality assessments completed at all three study timepoints

This was a secondary analysis of 149 women diagnosed with stage I-III BC randomized to 40 mg/day of atorvastatin or placebo (NCT01988571) in a 1:1 ratio in a double-blind fashion. This study was conducted through the Wake Forest National Cancer Institute Community Oncology Research Program (NCORP) Research Base (UG1CA189824) and was approved by the institutional review board at Wake Forest University School of Medicine (PREVENT-WF-98213) in accordance with the Declaration of Helsinki. The study enrolled participants from 31 community and academic centers across the United States from February 2014 through September 2020 (IRB approval date: 08/02/2013). Study visits took place prior to initiating cancer treatment and then at 6-months and 24-months from the start of cancer treatment. The aim of the parent study was to test if statin administration during receipt of anthracycline-based chemotherapy prevented declines in cardiac function. Primary results and full study protocol have been previously published [17]. Brief inclusion criteria were diagnosis of stage I–III BC or stage I–IV lymphoma, scheduled to receive adjuvant chemotherapy with an anthracycline, and at least 21 years of age. Brief exclusion criteria were use of a lipid-lowering agent within the last 6 months at screening, current post-menopausal hormone-replacement therapy, contraindication to undergoing magnetic resonance imaging, and liver disease, untreated hypothyroidism, inflammatory conditions, or unstable angina, ventricular arrythmias, or atrial fibrillation. All individuals provided written informed consent prior to participation. The present study included only female BC participants with muscle quality assessments (Fig. 1). Among BC participants, there were five individuals of Asian, Native Hawaiian, or Native American or Alaskan race. These individuals were also excluded from the analysis due to the small group sizes inhibiting valid statistical inference.

### Health-related Quality of Life (HRQoL)

HRQoL was measured using validated Patient-Reported Outcomes Measurement Information System (PROMIS®) questionnaires. HRQoL domains included cognitive function-abilities, depression, fatigue, pain interference, physical function, sleep disturbance, and ability to participate in social roles and activities. All PROMIS domains are scored using a T-score where a score of 50 is equivalent to the mean of the US general population and has a standard deviation of 10. A higher score for each measure indicates more of the concept measured. For example, a higher fatigue score means a greater level of fatigue whereas higher physical function means greater physical function capacity. PROMIS-Preference (PROPr) scores were calculated to combine HRQoL domains and yield a summary score. PROPr scores range from − 0.022–1.0, where 1.0 corresponds to a participant reporting full health and a high HRQoL, 0.0 corresponds to death, and scores below 0 represent the participant viewing their state as worth than death.

### Muscle quality

**Fig. 2:**
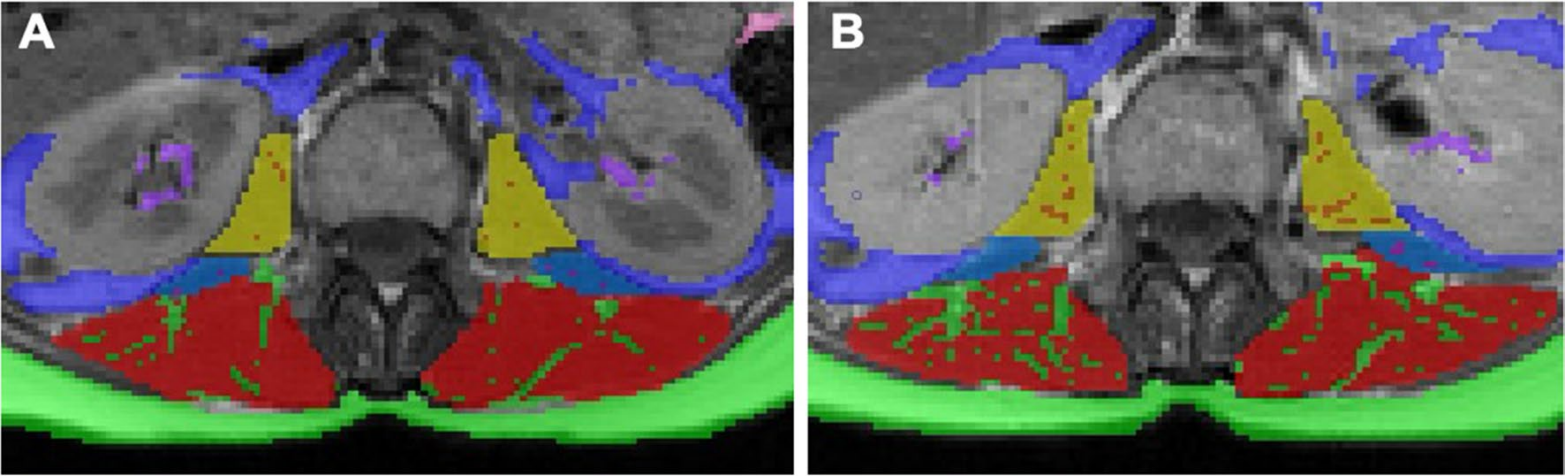
Paraspinal muscle quality segmentation**.** Example participant whose baseline IMF:SM increased from 0.099 (panel **A**) to 0.177 at 6 months (panel **B**). Paraspinal muscle quality was calculated by combining intermuscular fat area from the erector spinae (lime green within red), quadratus lumborum (pink), and psoas (orange) muscles and dividing it by the summed skeletal muscle area from the erector spinae (red), quadratus lumborum (blue), and psoas muscles (yellow)

Paraspinal muscle quality was measured using magnetic resonance imaging (MRI) as previously described [18, 19]. In brief, axial T1-weighted images were taken at the L2 vertebrae and analyzed for paraspinal skeletal muscle (SM) and IMF using SliceOmatic software (Tomovision) by a single technician blinded to the participant study group and study visit timepoint. IMF:SM ratio was calculated as the primary indicator of muscle quality. This ratio is used in heart failure and cardio-oncology populations to assess muscle quality and relates to clinical outcomes such as exercise capacity and cardiac function [19–22]. Figures 2a and b illustrate these readings from baseline and 6 months.

### Statistical analyses

Descriptive data are presented as mean ± standard deviation (SD) or count and percentage. Linear mixed-effects models with a patient-level random effect were used to describe muscle quality and HRQoL at baseline, 6 months, and 24 months. Study visit was treated as the main effect while adjusting for body mass index (BMI), study group (placebo or statin), age, and race (Black or White). An interaction term between study group and visit was used to examine if statin administration impacted changes in muscle quality. Potential confounders were evaluated via bivariate analyses to test if each variable was associated with the outcome and predictor. Covariates that were considered in the models but not included as they were not associated with the predictor and outcome were cancer stage, menopausal status, cumulative chemotherapy dose, adjuvant versus neoadjuvant therapy, hormone receptor status, anti-hypertensive medication use, smoking status, ethnicity, and income. Linear mixed-effects models with a patient-level random effect were also used to test if change in paraspinal muscle IMF:SM was associated with HRQoL and its domains, adjusting for the same covariates listed above. Linear mixed-effects models were used as they are robust to varying sample sizes at study timepoints. A two T-score point difference was interpreted to be a clinically meaningful group change for fatigue and physical function [23]. Analyses were also conducted using a median cut point (median = 0.199) for IMF:SM with participants categorized as having “low IMF:SM” or “high IMF:SM” and adjusted for the same covariates listed above. A meaningful individual change was defined as five T-score points, and participants were also categorized as meeting a clinically meaningful change of five points from baseline to 6-months [24]. Chi-squared tests evaluated differences in proportions of individuals with clinically meaningful changes. Post-hoc contrasts statements were used to compare PROPr scores, physical function, and fatigue levels at all study time points. Interaction terms were removed from models if the *p* value was > 0.2, otherwise, an alpha significance level was set at *p* = 0.05. All analyses were conducted in JMP Pro (Version 16, SAS, Inc., Cary, NC).

## Results

Baseline demographic and clinical characteristics are reported in Table 1. Average age of the cohort was 50.3 years old and participants were predominantly of White race (86%). Within this subset of the parent study, 54% were randomized to the statin group. Average BMI was near the obese category (29.9 kg/m^2^ ± 6.3), and 48% of the cohort reported a history of hypertension. Most of the participants (61%) were diagnosed with stage II breast cancer.

**Table 1:** Baseline demographic and clinical characteristics of women with breast cancer

Characteristic	N = 149
Age (years)	50.3 ± 10.7
Race	
White	128 (86)
Black	21 (14)
Study group	
Statin	81 (54)
Placebo	68 (46)
Body mass index (kg/m^2^)	29.9 ± 6.3
History of hypertension	71 (48)
Cancer stage	
I	18 (12)
II	91 (61)
III	40 (27)

Data are presented as mean ± SD or n (%)

Age missing, n = 19; body mass index missing, n = 19; hypertension medications missing, n = 23

**Fig. 3:**
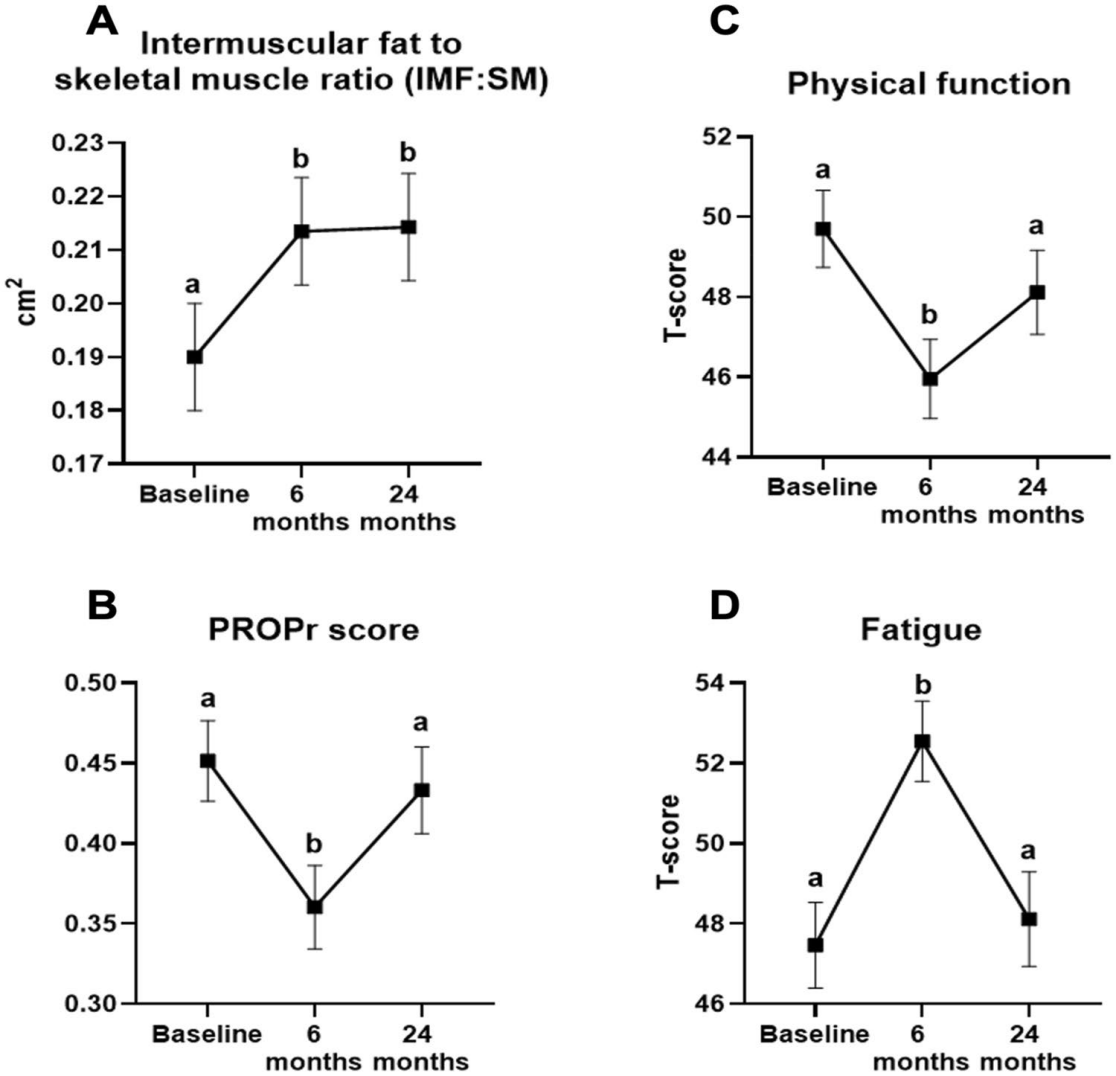
Intermuscular fat to skeletal muscle ratio (IMF:SM) and health-related quality of life measures. **A** IMF:SM increased from baseline (0.19 ± 0.01) to 6 months (0.213 ± 0.01, *p* < 0.001) and 24 months (0.214 ± 0.01, *p* < 0.001). **B** PROPr scores declined from baseline (0.45 ± 0.03) to 6 months (0.36 ± 0.03, *p* < 0.001) and returned to baseline levels at 24 months (0.43 ± 0.03, *p* = 0.62). **C** Physical function declined from baseline to 6 months (49.7 ± 0.96 vs. 45.95 ± 0.99, *p* < 0.001) and increased to 48.12 ± 1.1 (*p* = 0.15) at 24 months. **D** Fatigue levels increased (i.e., more fatigue) at 6 months (47.5 ± 1.1 baseline to 52.6 ± 1.0 at 6 months, *p* < 0.001) and declined to 48.1 ± 1.2 (p = 0.81) at 24 months. *p* Values are compared with baseline values. All PROMIS domains are scored using a T-score where a score of 50 is equivalent to the mean of the US general population with a standard deviation of 10. A higher score for each measure indicates more of the concept measured. Data points with different letters are statistically different at *p* < 0.05 by Tukey honestly significant difference tests. Adjusted means and standard errors are plotted. Covariates include age, race, body mass index, and study group (placebo or statin)

Muscle quality worsened over 24 months of cancer treatment, as shown by an increase in the IMF:SM ratio (Fig. 3A). SM did not change over the study period (*p* > 0.05), but IMF increased significantly at 6-months (*p* = 0.004) and 24-months (*p* = 0.007) relative to baseline (Table 2). Notably, changes in muscle quality during cancer treatment were independent of statin administration (study group-by-time interaction terms [β ± standard error]: − 0.007 ± 0.01, *p* = 0.63 for baseline-6 months; − 0.001 ± 0.02, *p* = 0.96 for 6-months-24 months).

**Table 2:** Adjusted means and standard error (SE) of skeletal muscle, intermuscular fat, and PROPr domains at baseline, 6 months, and 24 months

Domain	Baseline		6 months		24 months	
Muscle composition	Mean ± SE	Range	Mean ± SE	Range	Mean ± SE	Range
Skeletal muscle (cm^2^)	42.0 ± 0.8^a^	21.5–65.3	41.6 ± 0.8^a^	21.8–59.2	41.6 ± 0.8^a^	23.7–57.6
Intermuscular fat (cm^2^)	7.7 ± 0.4^a^	1.1–21.5	8.5 ± 0.4^b^	0.5–25.6	8.5 ± 0.4^b^	1.9–19.1
PROPr domains						
Physical function	49.7 ± 0.96^a^	28.9–57.0	45.95 ± 0.99^b^	26.6–57.0	48.12 ± 1.05^a^	22.5–57.0
Fatigue	47.47 ± 1.07^a^	33.7–75.8	52.55 ± 1.11^b^	33.7–75.8	48.12 ± 1.18^a^	33.7–75.8
Pain interference	52.06 ± 1.11^a^	41.6–75.6	53.23 ± 1.15^a^	41.6–75.6	51.79 ± 1.22^a^	41.6–75.6
Depression	51.01 ± 0.94^a^	41.0–73.3	49.12 ± 0.97^b^	41.0–79.4	49.4 ± 1.02^a,b^	41.0–71.2
Cognitive abilities	50.37 ± 1.07^a^	26.6–64.9	45.16 ± 1.11^b^	26.6–64.9	47.42 ± 1.19^b^	26.6–64.9
Social roles	54.47 ± 1.08 ^a^	27.5–64.2	51.74 ± 1.11^b^	27.5–64.2	53.8 ± 1.17^a,b^	27.5–64.2
Sleep	52.79 ± 0.88^a^	32.0–73.3	52.54 ± 0.91^a^	32.0–68.8	52.54 ± 0.97^a^	32.0–73.3

Data are presented as mean ± SE and are adjusted for age, race, body mass index, and study group (placebo or statin). Within each row, mean ± SE not connected by the same letter are statistically different at p < 0.05 by Tukey honest significant difference tests. Range for each variable are based on unadjusted values

HRQoL assessed by the PROPr score initially declined at 6 months but returned to levels similar to baseline at 24 months (Fig. 3B). A sensitivity analysis of participants with data at all three visits showed consistent results (supplemental Table 1). Physical function followed a similar pattern with declines from baseline to 6 months (mean difference ± standard error: 3.75 ± 0.8) but an improvement at 24 months (mean difference ± SE: 2.2 ± 0.9, Fig. 3C). Levels of fatigue increased (i.e., worsened) from baseline to 6 months (mean difference ± standard error: 5.1 ± 1.0) then lowered back to baseline levels by 24 months (mean difference ± standard error: 4.4 ± 1.1, Fig. 3D). The changes in physical function and fatigue represent clinically meaningful declines and recovery in these measures. Adjusted means, standard error, and ranges for each HRQoL domain are shown in Table 2. A score of 50 represents the average for U.S. populations.

### Muscle quality and health-related quality of life

**Fig. 4:**
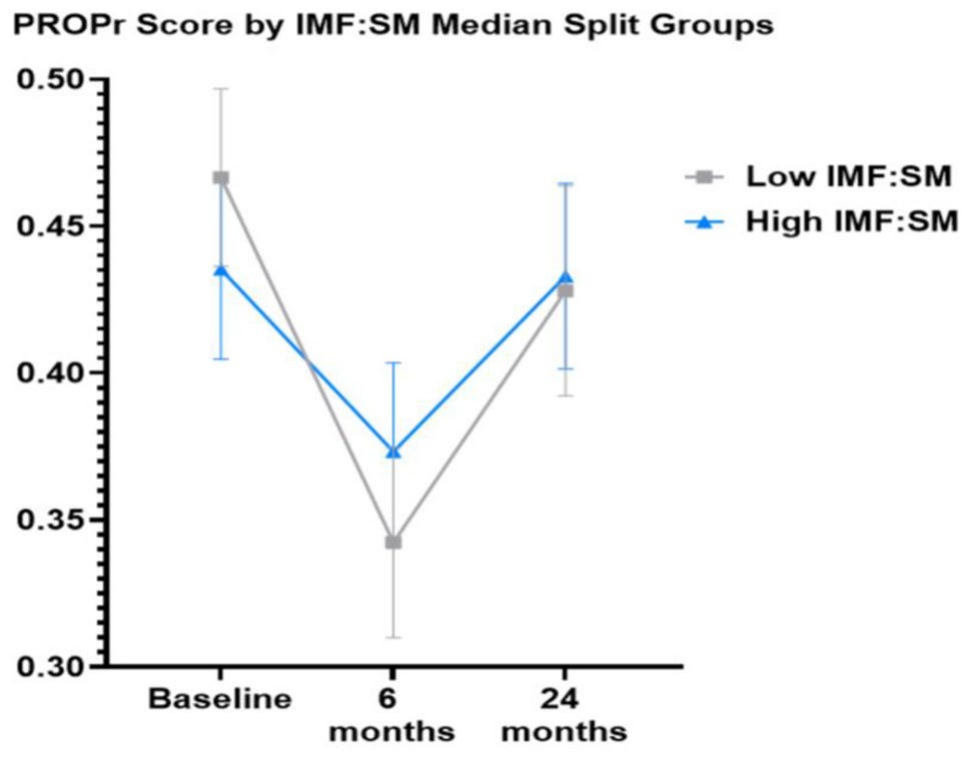
PROPr score over 24 months by paraspinal intermuscular fat to skeletal muscle (IMF:SM) ratio groups. Individuals with a lower baseline ratio of IMF:SM exhibited declines in PROPr scores from baseline to 6-months (− 0.12 ± 0.03, *p* < 0.001) whereas individuals with high IMF:SM at baseline did not report significant declines (− 0.06 ± 0.03, *p* = 0.14). Values at 24 months were similar to baseline for both groups (low IMF:SM group: 0.47 ± 0.03 vs. 0.43 ± 0.03, *p* = 0.82; high IMF:SM group: 0.44 ± 0.03 vs. 0.43 ± 0.03, *p* = 1.0)

Results of the mixed-effects models with paraspinal IMF:SM, PROPr score, physical function, and fatigue are shown in Table 3. There was a trend between paraspinal IMF:SM and PROPr score from baseline to 6-months (β estimate ± SE: 0.21 ± 0.11, *p* = 0.069). Individuals with better baseline muscle quality (i.e., low IMF:SM/below the median) experienced significant declines in PROPr score from baseline to 6-months (baseline to 6-months mean difference = − 0.12 ± 0.03, *p* < 0.001) that was double the decline of individuals with an IMF:SM value above the median at baseline (baseline to 6-months mean difference = − 0.06, *p* = 0.14, Fig. 4).

**Table 3:** Linear mixed-effects models relating paraspinal muscle quality (predictor) to PROMIS-Preference (PROPr) score for health-related quality of life, physical function, and fatigue levels

Predictor	PROPr score		Physical function	
	β estimate ± SE	*p* Value	β estimate ± SE	*p* value
Intercept	0.37 ± 0.11	0.001	55.6 ± 4.12	< 0.001
Paraspinal IMF:SM*24-month visit	− 0.07 ± 0.13	0.584	− 8.76 ± 5.79	0.132
Paraspinal IMF:SM*6-month visit	0.21 ± 0.11	0.069	7.01 ± 4.9	0.154
Paraspinal IMF:SM	− 0.13 ± 0.14	0.341	− 8.19 ± 5.55	0.141
Age	0.002 ± 0.002	0.318	− 0.03 ± 0.06	0.617
Black race	− 0.02 ± 0.02	0.385	− 0.34 ± 0.89	0.702
Body mass index	− 0.0002 ± 0.003	0.937	− 0.15 ± 0.09	0.115
24-month visit	0.02 ± 0.01	0.095	0.32 ± 0.51	0.536
6-month visit	− 0.05 ± 0.01	< 0.001	− 1.96 ± 0.48	< 0.001
Placebo group	0.01 ± 0.02	0.364	1.39 ± 0.6	0.021

Predictor	Fatigue			
	β estimate ± SE	*p* Value		
Intercept	52.97 ± 4.45	< 0.001		
Paraspinal IMF:SM	4.73 ± 6.12	0.441		
Age	− 0.11 ± 0.06	0.077		
Black race	− 1.05 ± 0.96	0.275		
Body mass index	0.03 ± 0.1	0.741		
24-month visit	− 1.36 ± 0.64	0.034		
6-month visit	3.15 ± 0.6	< 0.001		
Placebo group	− 0.59 ± 0.64	0.358		

Sample size at each timepoint: baseline, n = 127; 6 months, n = 112; 24 months, n = 85

There was a nonsignificant trend for worsening IMF:SM over 24 months and lower physical function scores (− 8.76 ± 5.79, *p* = 0.132). By median split IMF:SM, individuals with a low IMF:SM ratio at baseline and a high IMF:SM ratio both exhibited declines in physical function from baseline to 6 months (low IMF:SM mean difference = − 3.85 ± 1.19, *p* = 0.02 and high IMF:SM mean difference = − 3.57 ± 1.11, *p* = 0.02). Physical function values for both groups returned to levels similar to baseline values by 24 months despite worse muscle quality at 24 months. A similar proportion of participants reported a clinically meaningful change in physical function from baseline to 6-months for the low IMF:SM group and the high IMF:SM group (43% vs. 47%, respectively, *p* = 0.74). In models predicting fatigue, interaction terms between muscle quality and 6-month and 24-month study visits were not statistically significant and were removed from the models to interpret main effects (β ± SE: 5.0 ± 11.7, *p* = 0.67 and 2.0 ± 12.1, *p* = 0.87, respectively). Paraspinal IMF:SM was not associated with fatigue in this cohort (Table 3, *p* = 0.44). By median split groups, the low IMF:SM group reported similar levels of fatigue at all study time points. The high IMF:SM group showed an increase in fatigue levels from baseline to 6 months (mean difference: 6.0 ± 1.4, *p* < 0.001), which returned to levels similar to baseline by 24 months. A similar proportion of the low and high IMF:SM groups reported a clinically meaningful change in fatigue of at least 5 T-score points from baseline to 6-months (52% vs. 52%, *p* = 0.99).

Supplemental Table 3 displays the number and proportion of participants who reported clinically meaningful changes in fatigue and physical function across study time points. From baseline to 6-months, 55% of participants had a clinically meaningful decline in physical function whereas 30% had a clinically meaningful improvement in physical function from 6-months to 24-months (*p* < 0.001). By fatigue, 52% of participants reported clinically meaningful worsening of fatigue from baseline to 6-months, and 51% reported clinically meaningful improvements in fatigue from 6-months to 24-months (*p* = 0.22).

## Discussion

Both HRQoL and muscle quality worsened in the first six months of BC treatment, but by 24 months, HRQoL improved to levels similar to baseline whereas muscle quality continued to worsen. Individuals with better muscle quality at baseline reported greater declines in HRQoL from baseline to 6 months relative to those with worse baseline muscle quality. An increase in the IMF:SM ratio over 24-months was associated with a trend with lower physical function. These findings were independent of BMI and may indicate that preserving muscle quality during cancer treatment could support HRQoL in these patients.

In line with others, we report a decline in HRQoL in the first 6 months of active cancer treatment, but an increase and return to levels similar to baseline by 24 months [25]. Gao et al. also reported that global quality of life declined during receipt of treatment but improved following treatment and was comparable to a control population at 10 years of follow up [25]. Additionally, in the first 6 months, individuals with better baseline muscle quality reported twice the decline in PROPr scores relative to individuals with worse baseline muscle quality. At 24-months, both high and low IMF:SM groups reported HRQoL levels statistically similar to baseline. These findings were in spite of significant declines in muscle quality over the 24 months. This may indicate that patients adapt to a “new normal” following cancer treatment. This psychological concept was previously described as a shift in perception to a “new normal” as a positive predictor of quality of life outcomes following BC treatment [26–28]. Future studies that combine objective and subjective measures of HRQoL could explore this concept. As cancer survivorship continues to improve, there is increasing attention placed on patient-reported outcomes, such as HRQoL. These patient-reported HRQoL measures may capture symptoms experienced by patients that are unnoticed or not assessed by their physicians [7]. Findings here suggest that individuals who initiate cancer treatment with healthy muscle quality may be more likely to experience significant declines in HRQoL during treatment.

Muscle quality declined over 24 months, with the greatest declines occurring in the first six months. Changes in muscle quality were driven by increases in IMF over the study period rather than declines in SM. Additionally, statin administration did not impact changes in muscle quality over the study period. These findings confirm previous reports in a subset of this cohort and are in line with others showing an increase in fat mass without loss of lean mass [18, 29]. Decreased muscle quality correlates with increased inflammation, lower exercise capacity, impaired cardiac function, and higher mortality risk in cancer survivors [10, 15, 16, 18, 19]. We also examined predictors of muscle quality declines. We found baseline age and BMI were predictors of muscle quality, but statin administration, cancer stage, income, and anti-hypertensive medication use were not associated with changes in muscle quality. In other BC cohorts, little research has focused on predictors of muscle quality changes. One study that focused on total weight changes noted time since BC diagnosis, receipt of adjuvant therapy, African-American ethnicity, current energy intake, and postmenopausal status as predictors of body weight gain; whereas BMI, age at diagnosis, educational level, and exercise score were inversely associated with weight gain [30]. In the current cohort, physical activity and additional comorbid conditions were not assessed and may impact muscle quality. Additional research is needed to confirm predictors of muscle quality declines, which may identify targets for interventions that attenuate muscle quality declines.

We report a trend for poor muscle quality being associated with lower physical function over 24-months. Declines in physical function are one of the most common symptoms reported following cancer treatment, and cancer survivors have lower objectively-assessed physical function relative to age-matched controls [31, 32]. In this cohort, the self-reported changes in physical function in the first 6 months of treatment met thresholds for a clinically meaningful decline. A greater proportion of participants reported a clinically meaningful decline from baseline to 6-months (55%) than reported a clinically meaningful recovery in physical function from 6-months to 24-months (30%), indicating an attenuated recovery of physical function post-cancer treatment. Evidence also supports an accelerated age-related decline in physical function in cancer survivors [33]. In this cohort, cancer stage was not a significant predictor of physical function; however, others have reported that women with regional BC experience greater declines relative to women with local BC [33]. Factors driving decreased physical function are likely multifactorial, and findings reported here suggest that muscle quality may be one factor for consideration. Furthermore, decreased physical function and poor muscle quality are both independent predictors of mortality [14], and findings here suggest these factors may be correlated in women with BC. With a limited sample size at 24-months follow-up (n=85), the statistical trend noted between worsening muscle quality and physical function is worth further pursuit. Clinical trials are needed to determine if improving and/or preventing declines in muscle quality during cancer treatment can help avoid observed declines in physical function. Objective measures such as gait speed, handgrip strength, and chair stand tests may be simultaneously used in future research to compare with muscle quality.

Levels of fatigue worsened during active cancer treatment and returned to levels similar to baseline by 24 months follow up with changes in fatigue meeting thresholds for clinically meaningful changes. When examining median split groups, individuals with worse muscle quality reported a statistically significant increase in level of fatigue from baseline to 6 months while the group with better muscle quality did not, although both within group changes met thresholds for a clinically meaningful change of 2 points. As muscle quality was not associated with levels of fatigue in this cohort overall, the increase in levels of fatigue by the group with a higher IMF:SM ratio may reflect other characteristics of this group. While analyses were adjusted for age and BMI, there may be residual confounding or other characteristics that were not measured. For example, physical activity was not assessed in this cohort, but others have reported that higher physical activity is associated with better muscle quality in non-cancer groups and lower levels of fatigue in BC survivors [13, 34]. Further work is needed to understand associations between muscle quality and fatigue.

Physical activity interventions have shown improvements in HRQoL during and after BC treatment [35]. While physical activity was not assessed in this cohort, future research regarding impacts of physical activity on muscle quality should be investigated. Both exercise and diet modification can improve muscle quality [36, 37], but the optimal combination of aerobic and resistance training with or without caloric restriction or improvements to dietary quality for women treated for BC is not known.

Strengths of this study include the longitudinal design, including assessments taken pre-, during, and post-cancer treatment, and muscle quality determined by MRI. Limitations include the sample size and lack of data regarding total body composition or additional ectopic fat depots. Muscle quality assessment was limited to the paraspinal muscles, which is used to assess frailty in cancer populations [38], and has shown similar correlations to cardiorespiratory fitness as quadriceps [20]. However, measuring muscle quality in a larger group of working muscles, such as the quadriceps, should be considered and may yield different results. Physical activity and further assessment of comorbid conditions beyond hypertension were not available in this study and should be evaluated in future studies. Small sample sizes, particularly at follow-up timepoints, and limited enrollment of different racial groups inhibited our ability for statistical inference. Thus, these analyses included only women of White or Black race, which limits the generalizability of findings for other racial groups. HRQoL measures are self-reported patient assessments, and while insightful, pairing these assessments with objective measures in the future may be beneficial to confirm associations. Further research is also needed to determine whether declines in specific subdomains commonly associated with heart failure (HF), such as physical function, precede the onset of HF or correlate with stages of HF in BC survivors.

## Conclusions

Over 24-months of BC treatment, paraspinal muscle quality declined, predominantly due to increases in intermuscular adiposity. Declines in muscle quality trended with worse physical function at 24-months follow up. Individuals with better muscle quality beginning treatment may be at greater risk for declines in HRQoL. Larger studies are needed to confirm these findings and understand if improving muscle quality during BC treatment could help sustain HRQoL.

## Supplementary Information

Below is the link to the electronic supplementary material.

Supplementary file 1 (DOCX 18 KB)

## Data Availability

The datasets used and/or analyzed during the current study are available from the corresponding author on reasonable request.
