# Supplementary material for: Muscle quality during cancer treatment and associations with health-related quality of life
Source: Qual Life Res. 2026 Feb 12;35(3):71. doi: 10.1007/s11136-026-04175-2 (PMC12901223; doi:10.1007/s11136-026-04175-2)
Supplement: Supplementary file 1 — (DOCX 18 KB) [file 11136_2026_4175_MOESM1_ESM.docx]

**SUPPLEMENTAL MATERIAL:** **Muscle Quality During Cancer Treatment and Associations with Health-Related Quality of Life**

**Table of Contents**

[Supplemental Table 1 2](#_Toc165379079)

Supplemental Table 2…………………………………..………………………………………….3

Supplemental Table 3……………………………………………………………………………...4

| **Supplemental Table 1. Sensitivity analysis of PROPr scores over the study time period in participants with data available at all study visits.** | | | |  |
| --- | --- | --- | --- | --- |
| **Term** | **Estimate** | **Std Error** | **Prob>\|t\|** |  |
| Intercept | 0.45 | 0.19 | 0.0207 |  |
| Visit (Baseline-24Months] | 0.007 | 0.02 | 0.78 |  |
| Visit (Baseline-6 Months) | -0.09 | 0.02 | 0.001 |  |
| Black Race | 0.03 | 0.09 | 0.73 |  |
| Body mass index | 0.001 | 0.004 | 0.84 |  |
| Age | -0.001 | 0.003 | 0.68 |  |
| Placebo | 0.08 | 0.05 | 0.12 |  |

| **Supplemental Table 2. Sensitivity analysis in participants with complete data available at all study visits of changes in paraspinal muscle quality and PROPr scores (outcome) over the study time period.** | | | |
| --- | --- | --- | --- |
| **Term** | **Estimate** | **Standard error** | **p-value** |
| Intercept | 0.524 | 0.193 | 0.0087 |
| Visit (24-months – baseline) | 0.001 | 0.025 | 0.9675 |
| Visit (6-months – baseline) | -0.084 | 0.024 | 0.0008 |
| Black race | 0.019 | 0.089 | 0.8289 |
| Body mass index | 0.002 | 0.004 | 0.6759 |
| Age | 0.000 | 0.003 | 0.9038 |
| Placebo group | 0.076 | 0.050 | 0.1357 |
| Paraspinal IMF:SM | -0.608 | 0.302 | 0.0461 |
| Paraspinal IMF:SM*24-month visit | 0.323 | 0.303 | 0.2891 |
| Paraspinal IMF:SM*6-month visit | 0.823 | 0.300 | 0.0071 |

| **Supplemental Table 3. Number and proportion of participants who reported a clinically meaningful change of 5 T-score points in physical function and fatigue domains** | | | |
| --- | --- | --- | --- |
|  | Baseline to 6-month changes | 6-month to 24-month changes | p-value |
| Physical function | 49 (55) | 21 (30) | <0.001 |
| Fatigue | 47 (52) | 37 (51) | 0.22 |
| Count and percentage for each cell are displayed. P-values compare the proportion within each domain. | | | |
